# Data integrity of 14 randomised controlled trials

**DOI:** 10.1016/j.ejogrb.2024.05.007

**Published:** 2024-05-10

**Authors:** Ben W Mol, Esmee M Bordewijk, Ewelina Rogozińska, Lyle C Gurrin, Jim G Thornton, Madelon van Wely

**Affiliations:** 1 Department of Obstetrics and Gynecology, Monash University, Clayton, Australia; 2 Centre for Reproductive Medicine, Amsterdam UMC, Amsterdam, The Netherlands; 3 Meta-Analysis Group, Institute of Clinical Trials and Methodology, MRC Clinical Trials Unit at UCL, London, England, United Kingdom; 4 Centre for Epidemiology and Biostatistics, School of Population and Global Health, The University of Melbourne, Parkville, Australia; 5 Faculty of Medicine & Health Sciences, University of Nottingham, Nottingham, United Kingdom

## Abstract

**Background:**

A review of the literature on iron treatments for iron-deficient anaemia in pregnancy indicated duplication of baseline and outcome tables in two separate randomised controlled trials (RCTs) that share only a single author.

**Aim:**

To assess the integrity of randomised clinical trials from Dr A.M. Darwish, Assiut University, Egypt.

**Design:**

Assessment of Research Integrity.

**Methods:**

We tabulated the characteristics of studies, compared baseline and outcome tables between articles and looked for implausible findings. We used the distribution of baseline p-values to assess whether the summary statistics of baseline characteristics were consistent with properly conducted randomisation.

**Results:**

We identified 14 RCTs (1,405 participants) published between October 2004 and September 2019. Two pairs of studies showed considerable similarities in baseline characteristics, while another pair of studies was plagiarized. The analysis of baseline p-values indicated a low probability that all the studies featured randomised treatment allocation.

**Conclusion:**

Our analysis of the RCTs of Dr Darwish suggests possible integrity problems. We recommend a critical investigation of the studies that have not been retracted. Until that has been completed, these studies should not be used to inform clinical practice.

## Introduction

A review of the literature of iron treatments for iron-deficient anaemia in pregnancy indicated integrity problems in two randomised controlled trials (RCTs) from the author Dr A.M. Darwish, Assiut University, Egypt (Dawish 2017, Darwish 2019a0 (1). There were worrying similarities between the tables of papers that purport to present the results of distinct studies. We therefore assessed all the papers of this author, following methods as previously described (2).

## Materials and Methods

### Inclusion of RCTs

We searched the PubMed database and Google for authors ‘Darwish’ using the affiliation ‘Assiut’ restricting to RCTs.

### Data extraction

We extracted information regarding year of publication, journal, study centers, baseline characteristics, number of participants, outcome data, study start and end dates, and date of submission to the journal. We calculated the average number of randomised participants per month for each study by dividing the total number of randomised participants by the number of months over which recruitment was said to have occurred. We obtained the trial registration numbers by searching the World Health Organization (WHO) and International Standard Randomised Controlled Trial Number (ISRCTN) registers. We also reviewed the studies for clinical findings and consistency of the methods and results.

### Comparison of baseline characteristics and outcomes

We performed pairwise comparisons of the values in the baseline characteristics and outcome tables to find identical or similar values across trials. We compared values of mean, standard deviation (SD), percentage, *t*-value, *p*-value, and confidence intervals (CIs) whenever available.

### Probability of random sampling for baseline characteristics

The distribution of *p*-values comparing baseline variables between groups in RCTs has been used successfully to assess data integrity (3 4). These earlier works assumed that, if randomisation has been done reliably, that baseline p-value distributions from continuously-valued variables are uniformly distributed on the interval (0,1). However, this assumption is challenged by potential correlations between baseline variables, different randomisation methods, and rounding of summary statistics (5). Recently, the empirical distribution of baseline p-values was generated using data of a large number of authentic RCTs (Bolland 2020). In this analysis, we used this empirical distribution rather than uniform distribution as the reference to overcome the limitations of earlier methods.

We compared the baseline variables between trial arms with a *t*-test or one-way ANOVA using mean and standard deviation reported in trial publications. We used a Kolmogorov–Smirnov test, with the empirical distribution as the reference, to assess quantitatively the evidence against the null hypothesis that baseline characteristics in RCTs of Dr. Darwish were results of a robust randomisation process. Statistical analyses were performed using Stata (v16.0) and the R statistical software (v3.5.1).

## Results

### Inclusion of RCTs

We identified 14 RCTs (1,405 participants) published between October 2004 and September 2019. Dr.Darwish was the first author in 13 trials and co-authored one. The median number of participants per study was 112 (range 27 to 256), with a median number of participants per month of 5 (range 1 to 8). Five RCTs were retrospectively registered, nine unregistered, and none prospectively registered (Table 1, Table 2).

### Comparison of baseline characteristics and outcomes of the RCTs of A.M. Darwish

**Figure 1:**
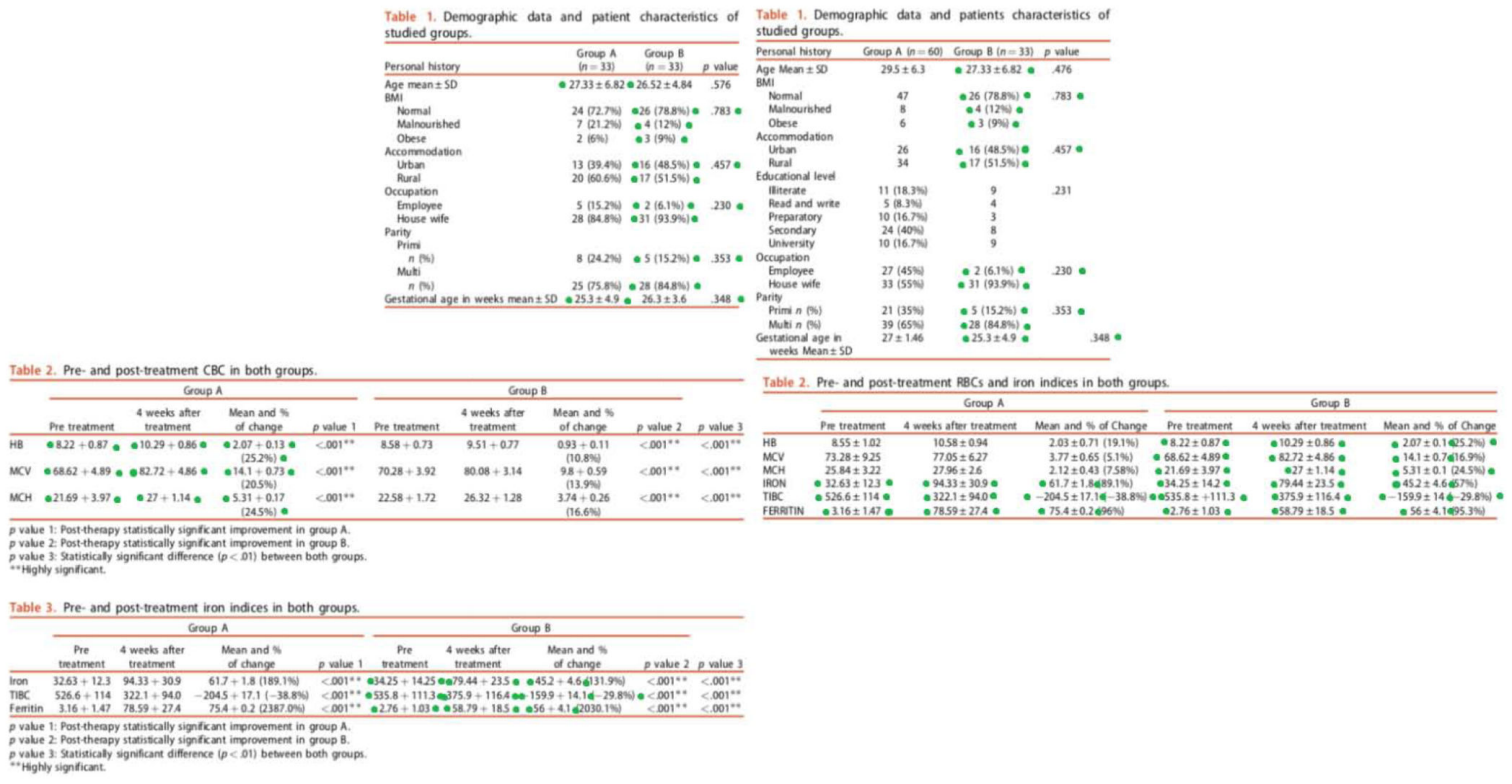
Similarities between Darwish 2019a and Darwish 2017 Darwish 2017 (table on the left) and Darwish 2019a (table on the right) both published in J Matern Fetal Neonatal Med. Both retracted 30 months after the first concerns were raised. Green dots represent the exact same number.

We found that the study of Darwish 2019a published in *The Journal of Maternal-Fetal & Neonatal Medicine* had 27 values in the baseline table and 38 values in the outcome table that were identical to the values in the corresponding table of Darwish 2017 that was published in the same journal (Figure 1). In Darwish 2017, 66 anaemic pregnant women were randomised to receive low molecular weight iron dextran as a total dose infusion or oral iron ferrous fumarate. In Darwish 2019a 120 pregnant women with gestational age greater than 14 weeks with confirmed clinical and laboratory evidence of iron deficiency anaemia were randomised to receive oral lactoferrin plus health education provided by a nurse versus total dose infusion of low-molecular weight iron dextran. Darwish 2017 was reported to have been conducted between March 2015 and February 2016, while Darwish 2019a between September 2015 and October 2017. Both studies have been retracted by the *The Journal of Maternal-Fetal & Neonatal Medicine*.

**Figure 2:**
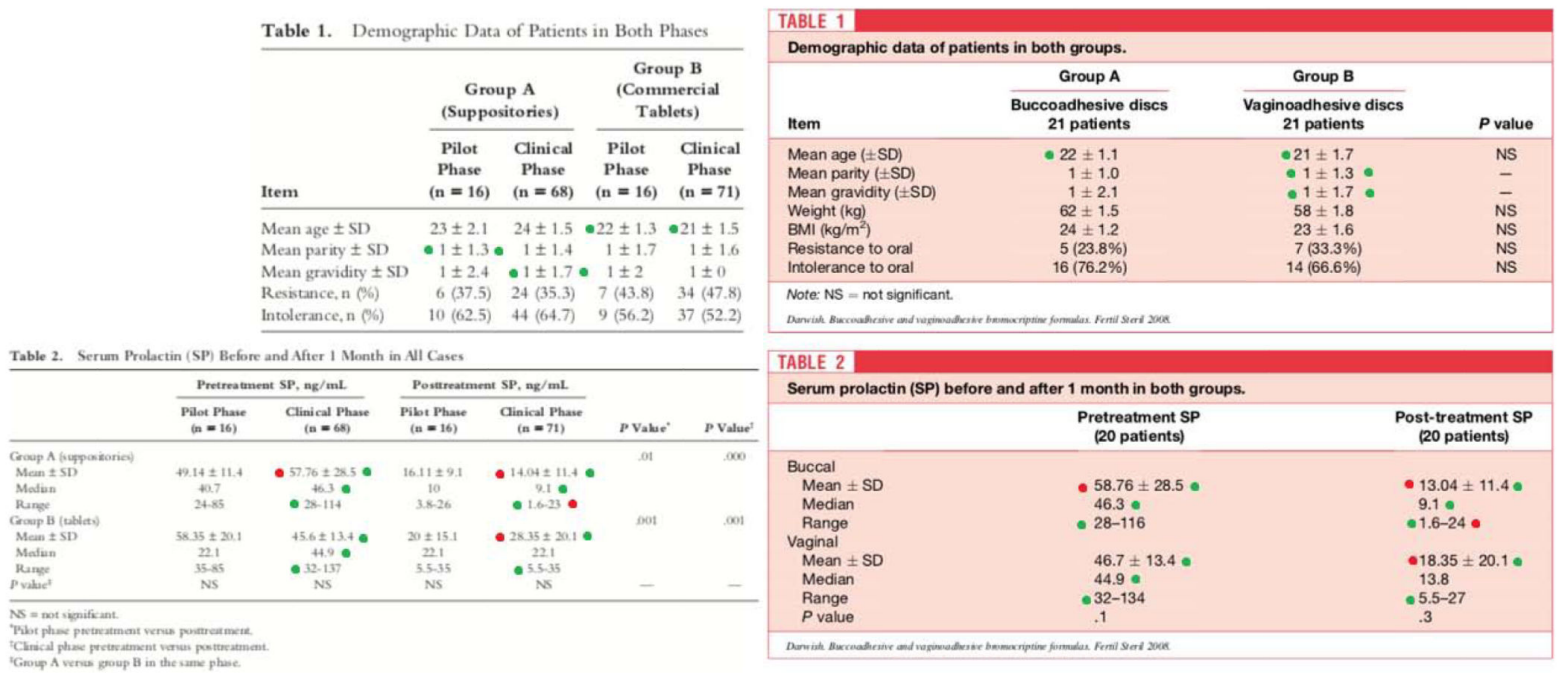
Similarities between Darwish 2007b and Darwish 2008 Darwish 2007b (table on the left) was published in *Reproductive Sciences*. Darwish 2008 (table on the right) was published in *Fertility and Sterility*. Green dots represent the exact same number and the red dots represents -1 or +1 for any digit.

We also found that Darwish 2007b and Darwish 2008 had 17 similar values and four very similar values (+1 or -1) in the baseline characteristics and outcome tables (Figure 2). Darwish 2007b reports on 171 patients with hyperprolactinemia. A pilot phase comprised 32 patients who were divided into 2 groups. Group A comprised 16 patients who used vaginal suppositories containing 2.5 mg bromocriptine mesylate with pluronics and bioadhesive agents once daily for 1 month, while group B included 16 patients who used commercial 2.5-mg bromocriptine mesylate tablets (mis-spelt as “mesthylate” in the article) inserted vaginally once daily for 1 month. The clinical phase comprised 139 patients who were again divided into 2 groups in the same way (group A, 68 patients; group B, 71 patients).

Darwish 2008 reports on 42 patients with pathologic hyperprolactinemia randomised to receive unidirectional bucco-adhesive bromocriptine mesylate discs or vagino-adhesive bromocriptine mesylate discs. Darwish 2007b was reported to have been conducted between September 2004 and August 2006, while Darwish 2008 between April 2004 to March 2007.

**Figure 3:**
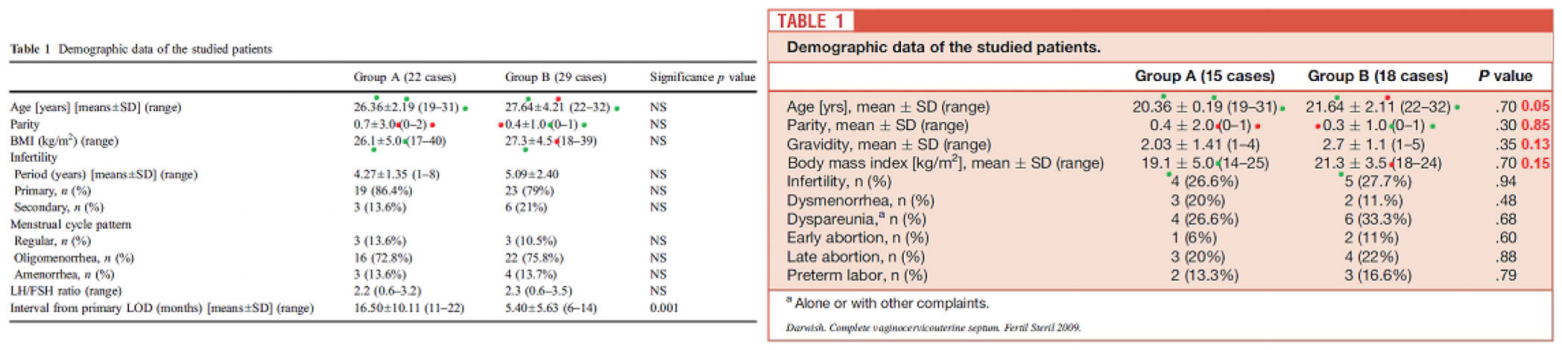
Similarities between an observational study and Darwish 2009 The table on the left shows the demographic data of an observational study of Darwish which was published in *Gynaecology surgery* in 2009. The article was submitted on 15 march 2008. Darwish 2009 (table on the right) was published in *Fertility and Sterility*. The article was received on 30 April 2008. Green dots represent the exact same number and the red dots represents -1 or +1 for any digit.

The paper of Darwish 2019b was almost identical to a paper of Kamel 2019 on which Darwish was co-author. Both papers were published in 2019, Darwish 2019b in the *Journal of Mid-life Health* under the title: “Office Cervicoscopy versus Stationary Colposcopy in Suspicious Cervix: A Randomized Controlled Trial.”, and Kamel 2019 in the *Journal of Current Medical Research and Practice* with the title: “Office Cervicoscopy versus Stationary Colposcopy in cases with Suspicious Cervix: A Randomized Controlled Trial.” The four authors are the same, with Darwish and Kamel swapping places between the two papers. Study period (July 2016 – December 2017) and number of participants (112 randomised out of 250 eligible) were identical. Figures 1, 2 and 3 were identical, with Table 3 (Darwish 2019b) and Table 4 (Kamel 2019) differing in only one line. The conclusion that cervicoscopy is a good complementary tool for detection of cervical lesions was worded differently, but virtually the same. A plagiarism scan showed 41% similarity. Both papers are currently retracted.

### Irregularities

Darwish 2004 compared intravaginal misoprostol versus endocervical laminaria tents prior to operative hysteroscopy, and reported misoprostol to be superior. The study was described as a double-blind study but it is unclear how this was achieved without the use of placebo.

Darwish 2007a reported an RCT with four antibiotic regimens in pregnant women with bacterial vaginosis. The authors reported detailed variables like changes in Amsel’s criteria, nausea, metallic taste, loose stools and rashes without one missing value in 156 women over 4 years. Mean age has values of 22, 23, 25 and 26 and gestational age at intervention 29, 30, 32 and 33. The number of women with preterm rupture of the membranes (PROM) in the 4 groups was 20, 21, 22, and 23 while it was 16, 17, 18, and 19 in the women with threatened preterm labour. The standard deviation for prolongation of pregnancy has values between 2 and 4, which is counterintuitive. It is also remarkable that in 4 groups of randomised women the gestational age at inclusion varied between 29 weeks versus 33 weeks, but this distribution of values was reported as not-significantly different.

Darwish 2007b reported on a randomised trial comparing vaginal bioadhesive suppositories as compared with vaginal use of commercial bromocriptine tablets in hyperprolactinemic patients. Mean age had values 21, 22, 23 and 24, with the two biggest groups of 68 and 71 women remarkably different (Supplementary Table 1). Gravidity and parity were exactly the same (indicating no previous miscarriage) and a mean value of 1 is high for an infertile population. Standard deviations for parity have values 1.3, 1.4, 1.6 and 1.7, while for gravidity they are reported to be 0 and 2.

Darwish 2009 reported on a randomised trial comparing extended resectoscopic versus sequential cold knife–resectoscopic excision of the unclassified complete uterocervicovaginal septum. While mean gravidity at baseline was 2.0 (N=15) and 2.7 (N=18), mean parity was 0.3 and 0.4, the number of women with an ‘early abortion’ was only 1 and 2, respectively. We found that a comparative study submitted 6 weeks earlier than Darwish 2009 has 10 similar values and five very similar values (+1 or -1) in the baseline characteristics. We could not reproduce the *p*-values of Darwish 2009 (Figure 3). Also, looking at the baseline characteristics Infertility, Dysmenorrhea, Dyspareunia, Early abortion, Late abortion, and Preterm labour, the number of women in group A are 1, 2, 3, and 4, and in group B are 2, 3, 4, 5, and 6.

Darwish 2010 compared bipolar versus monopolar resectoscopic myomectomy. The last digits of Serum potassium concentration are 6, 7, 8, and 9 in group A and 6, 7, and 8 in group B.

Darwish 2018 reported on a randomised controlled trial comparing Bakri balloon versus condom-loaded Foley’s catheter for treatment of atonic postpartum hemorrhage. Between October 2014 and December 2015 there were 100 women eligible with post-partum hemorrhage not responding on medical treatment, of whom 66 were randomised. Despite postpartum hemorrhage being an acute clinical situation, none of the women was excluded because of a lack of time. The amount of blood loss was not reported.

### Probability of random sampling for baseline characteristics

**Figure 4:**
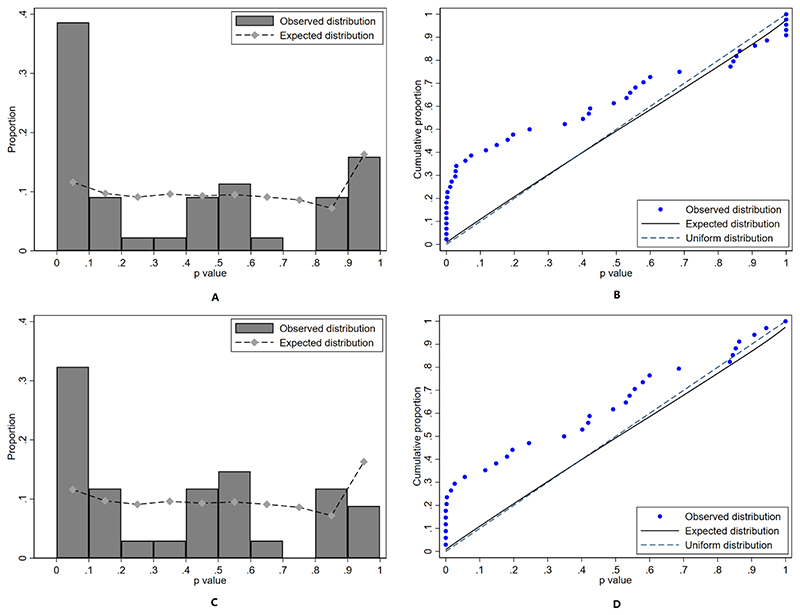
Distribution of baseline p values for continuous characteristics from RCTs by Darwish. 4A shows the histogram all RCTs 4B shows the cumulative function of all RCTs; 4C, 4D show the histogram and cumulative function of 8 RCTs in which we could not find mutual similarities The null hypothesis is that the baseline characteristics in intervention and controls groups in these RCTs are the results of a properly conducted randomization process. The distribution was inconsistent with the expected distribution suggesting these baseline characteristics are not generated from proper randomisation.

For the 13 trials of which Darwish is the leading author, the distribution of baseline variables substantially violated the empirical distribution (*p*-value=0.0006767), which also suggests a low probability that all these baseline characteristics are the results of proper randomization (Figure 4A & 4B). When we repeated the analysis after excluding the RCTs that had similar baseline and outcome analyses, the baseline *p*-values still violated the empirical distribution (p-value=0.02007, Figure 4C & 4D).

## Discussion

We examined 14 RCTs published between October 2004 and September 2019 in 11 different journals, of which Dr Darwish was first author 13 times and co-author once. Our assessment shows similarities in baseline and outcome tables in two pairs of two articles, one double publication, and similarities in the baseline table between an observational study and RCT. Analysis suggested that the observed distribution of baseline variables would occur with a low probability if treatment allocation in these studies is truly randomised. Assessment of the individual studies showed a series of implausible findings, including unlikely recruitment in acute situations (Darwish 2018), unlikely completeness of data (Darwish 2007a) and patterns of numbers in tables that are not consistent with the effect of chance (Darwish 2007a, Darwish 2007b, Darwish 2009).

Since our analysis, we have written to editors about 10 of RCTs. Five of his papers have been retracted (Darwish 2016, Darwish 2017, Darwish 2019a, Darwish 2019b, Kamel 2019), one 4 months after flagging the concern, one after 6 months and 2 after 30 months. Six other journals are still investigating after more than 12 months.

We wrote to Dr Darwish and his Institute Assiut University in September 2020. Dr Darwish informed us that all data were lost, and that as busy clinicians there was no further time to respond. We never received a response from Assiut University. Assiut University has by means of their human research ethics committee (head Dr Abdel Aleem, a co-author on the *Gynecological Surgery* 2009 paper, Figure 3) promised to investigate other problematic authors, but we have not been made aware of the results of any such investigations (Bordewijk 2021).

Our analysis suggests that editors should be reluctant to accept retrospectively registered RCTs. Without adequate evidence of, for example, an ethics approval, that should have in and of itself prevented publication. Based on our findings, we suggest a critical investigation of the non-retracted Darwish papers, including the non-randomised studies. We propose that the involved editors collaboratively ask for original individual participant data, and official documents such as study protocols, statistical analysis plans and letters confirming ethical approval by an institutional panel. We feel, in the face of so many irregularities, that such an investigation should also include other non-randomised publications by this author.

In conclusion, our analysis of the 14 RCTs of Dr. Darwish suggests integrity problems. We advise a critical investigation of the studies that have not been retracted, ideally facilitated by collaboration between all the involved editors and publishers. Until that has been completed, the studies of Dr. Darwish should not be used to inform clinical practice.

## Figures and Tables

**Table 1:** Fourteen randomised clinical trials of Darwish

Study	Journal	Title	Written to the editor	Retracted
Darwish 2004	Human Reproduction	Cervical priming prior to operative hysteroscopy: a randomized comparison of laminaria versus misoprostol.	Insufficient evidence^*^	
Darwish 2007a	Journal of Obstetrics and Gynaecology Research	Treatment options for bacterial vaginosis in patients at high risk of preterm labor and premature rupture of membranes	23/02/22	
Darwish 2007b	Reproductive Sciences	Superiority of Newly Developed Vaginal Suppositories Over Vaginal Use of Commercial Bromocriptine Tablets: A Randomized Controlled Clinical Trial	24/02/22	
Darwish 2007c	Acta Obstetricia Gynecologica Scandinavia	Is there a role for hysteroscopic tubal occlusion of functionless hydrosalpinges prior to IVF/ICSI in modern practice?	Insufficient evidence^*^	
Darwish 2008	Fertility and Sterility	Clinical efficacy of novel unidirectional buccoadhesive vs. vaginoadhesive bromocriptine mesylate discs for treating pathologic hyperprolactinemia	24/02/22	
Darwish 2009	Fertility and Sterility	Extended resectoscopic versus sequential cold knife–resectoscopic excision of the unclassified complete uterocervicovaginal septum: a randomized trial	25/02/22	
Darwish 2010	J. Obstet. Gynaecol. Res.	Biological effects of distension media in bipolar versus monopolar resectoscopic myomectomy: A randomized trial	23/02/22	
Darwish 2013	Journal of Lower Genital Tract Disease	Trichloroacetic Acid Application Versus Spray Monopolar Diathermy for Treating Benign Cervical Lesions: A Randomized Controlled Clinical Trial	Insufficient evidence^*^	
Darwish 2016	Gynecological Surgery	Monopolar versus bipolar laparoscopic ovarian drilling in clomiphene-resistant polycystic ovaries (PCO): a preliminary study	26/02/22	Sept 22
Darwish 2017	The Journal of Maternal-Fetal & Neonatal Medicine	Total dose iron dextran infusion versus oral iron for treating iron deficiency anemia in pregnant women: a randomized controlled trial	22-04-20	Dec 22
Darwish 2018	The Journal of Maternal-Fetal & Neonatal Medicine	Bakri balloon versus condom-loaded Foley’s catheter for treatment of atonic postpartum hemorrhage secondary to vaginal delivery: a randomized controlled trial	17-03-22	
Darwish 2019a	The Journal of Maternal-Fetal & Neonatal Medicine	Lactoferrin plus health education versus total dose infusion (TDI) of low-molecular weight (LMW) iron dextran for treating iron deficiency anemia in pregnancy: an RCT	22-04-20	Dec 22
Darwish 2019b	Journal of Mid-life Health	Office Cervicoscopy versus Stationary Colposcopy in Suspicious Cervix: A Randomized Controlled Trial	27-08-20	Jan 21
Kamel 2019	Journal of Current Medical Research and Practice	Office cervicoscopy versus stationary colposcopy in cases with suspicious cervix: a randomized controlled trial	27-08-20	Nov 20

* Insufficient evidence refers to the fact that based on the findings in the individual paper there was insufficient evidence to write to the journal.
